# Clinical implications of a novel SERPINA1 variant c.236 T > A: Challenges in characterizing new rare alpha-1 antitrypsin mutations

**DOI:** 10.1016/j.ymgmr.2026.101295

**Published:** 2026-02-13

**Authors:** Arturo Olivares-Rivera, Hilal Ersöz, Philipp Höger, Martina Veith, Timm Greulich, Kai Schlamp, Sabina Janciauskiene, Felix Herth, Franziska C. Trudzinski

**Affiliations:** a Department of Pneumology and Critical Care Medicine, Thoraxklinik, University of Heidelberg, Heidelberg, Germany; b Translational Lung Research Center (TLRC-H), German Center for Lung Research (DZL), Heidelberg, Germany; c University Medical Center Giessen and Marburg, Philipps-University, Department of Medicine, Pulmonary and Critical Care Medicine, Member of the German Center for Lung Research (DZL), Marburg, Germany; d Department of Diagnostic and Interventional Radiology with Nuclear Medicine, Thoraxklinik at University Hospital Heidelberg, Heidelberg, Germany; e German Center for Lung Research (DZL), Biomedical Research in End-Stage and Obstructive Lung Disease Hannover (BREATH), Hannover, Germany

**Keywords:** Emphysema, Alpha-1 antitrypsin deficiency, SERPINA1, Case report, Rare mutation

## Abstract

Severe alpha-1 antitrypsin deficiency (AATD) is a rare genetic condition characterized by low levels of alpha-1 antitrypsin (AAT), leading to progressive lung and/or liver disease. Most severe cases are linked to the Z allele (c.1096G > A (p.Glu366Lys)) of the *SERPINA1* gene but characterizing patients with rare mutations remains challenging. This case report discusses the clinical significance of a novel *SERPINA1* variant, c.236 T > A (p.Val79Glu; ClinVar accession SCV007334878), and the challenges in profiling such cases. Two male siblings carried both the common Z allele mutation and the novel exon 2 mutation. Despite genetic similarities, their clinical courses diverged. The older brother, a 66-year-old patient, presented with very severe airflow obstruction (GOLD stage IV) and an AAT level of 0.32 g/L. After years of pharmacologic treatment and endoscopic lung volume reduction, his condition worsened, requiring a double lung transplant. In contrast, the younger brother, currently 58 years old, was diagnosed through family screening and had an AAT level of 0.4 g/L. His condition progressed to panlobular basal emphysema accompanied by bronchopathy and mild bronchiectasis, managed with pharmacologic therapy. Both siblings had a history of smoking, potentially influencing their clinical outcomes. This case highlights the complexity of assessing rare *SERPINA1* mutations due to underlying biochemical complexities, genetic variability, and diagnostic limitations. It underscores the importance of combining biochemical analysis of variant AAT proteins with clinical evaluation to better understand disease expression, the value of genetic screening in family members, and the need for personalized clinical management to support timely and appropriate therapeutic interventions.

## 1 Background

Alpha-1 antitrypsin (AAT) is an acute-phase glycoprotein primarily produced in the liver, released into the bloodstream, and transported to the lungs through the vascular system [1]. Due to its serine protease inhibitory activity [2,3], AAT serves as a potent regulator of neutrophilic-derived proteases, particularly neutrophil elastase (NE) [4,5], thereby protecting lung tissue from excessive degradation during inflammatory responses [6]. Neutrophils and NE play an important role in the lung's immune defense against external noxae, such as microbial infections [7,8] or exposure to harmful substances from air pollution and cigarette smoking [9]. In addition to its antiprotease activity, AAT also exerts important immunomodulatory and anti-inflammatory effects through interactions with cytokines, immune cells, and metabolic pathways, highlighting its broader role in immune homeostasis [[10], [11], [12]].

Severe alpha-1 antitrypsin deficiency (AATD) is a rare genetic condition characterized by a marked reduction (up to 90%) or absence AAT protein in patient's serum [13,14], leading to variable clinical manifestations, including progressive lung disease and, in some cases, liver involvement [15]. Cigarette smoke can further impair AAT function [16], accelerating the onset and progression of lung disease in AATD patients [5]. This is often related to the development of chronic obstructive pulmonary disease (COPD), bronchiectasis, and emphysema. Despite the availability of specific augmentation therapy with plasma purified AAT, many AATD patients are diagnosed too late due to the rarity of the condition, its variable clinical presentation, and a general lack of awareness among healthcare professionals. In severe cases, delayed diagnosis may result in disease progression to end-stage lung failure, ultimately requiring lung transplantation.

The majority of severe AATD cases are linked to the Z allele mutation (c.1096G > A (p.Glu366Lys); rs28929474) in the SERPINA1 gene, which accounts for over 95% of diagnosed cases [17]. However, clinical characterization of patients carrying rare SERPINA1 variants remains challenging, as current diagnostic algorithms are primarily designed to detect the most common mutations. This is particularly important given that more than 500 variants have been identified to date [18,19], and emerging evidence suggests that some rare mutations may pose a higher risk for severe AATD-related symptoms than previously recognized [20].

This case report discusses the challenges in clinical profile characterization of two male siblings who share a newly identified rare mutation but had strikingly different clinical courses.

## 2 Case presentation

**Fig. 1:**
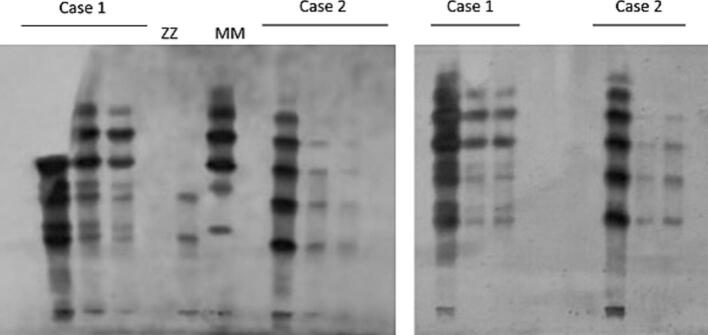
Isoelectric focusing (IEF). Isoelectric focusing (IEF) of serum alpha-1 antitrypsin (AAT). Reference phenotypes Pi*ZZ and Pi*MM show the expected slow-migrating Z pattern and sharp mid-gel M bands, respectively. Samples from Case 1 and Case 2 display a Z-type band together with a second band migrating in the M-range. SERPINA1 sequencing identified heterozygosity for c.1096G > A (p.Glu366Lys) and a novel exon 2 variant, c.236 T > A (p.Val79Glu). Despite M-like mobility, low serum AAT levels indicate a functionally deficient pseudo-M isoform.

The two patients described in these case reports presented to the Special Outpatient Department of the Thoraxklinik Heidelberg due to their COPD. As part of the diagnosis, AAT serum level was measured using nephelometry, and genotyping was performed with the Progenika A1AT genotyping kit (Progenika Biopharma, S.A., Derio, Spain) using the Luminex 200 system (Luminex, Austin, TX, USA). In this approach, polymerase chain reaction (PCR) amplification is followed by hybridization of PCR products to allele-specific probes immobilized on color-coded microspheres, enabling simultaneous detection of the 14 most prevalent mutations of the *SERPINA1* gene, which are commonly associated with AATD. The PCR was followed by isoelectric focusing (IEF) (Hydrasis 2 scan focusing, Sebia, Fulda, Germany) with PIM and PIZ reference standards; bands were interpreted according to laboratory SOPs and established migration criteria (Fig. 1). Both methods were performed by the AAT laboratory of the University Hospital of Marburg. In the case of conflicting results between PCR and IEF diagnostics, or in patients with an indication of a rare mutation, sequencing of all coding exons and exon–intron boundaries of the *SERPINA1* gene by Next Generation Sequencing was carried out as standard (Progenika Biopharma, Spain, Next Generation Sequencing). High-resolution computed tomography of the chest (HRCT) scans were performed on both patients and analyzed using the software YACTA to detect and quantify emphysema on consecutive thin-slice HRCT datasets. YACTA provides visualization of the affected areas of emphysema using color overlays on the original HRCT images.

The two brothers described in this report both carried the heterozygous *Z*-allele mutation (c.1096G > A (p.Glu366Lys); rs28929474) and a heterozygous additional, previously undescribed mutation in exon 2 (note: exons 1 A, 1B, and 1C are non-coding 5′UTR exons; exon 2 is the first coding exon), position c.236 T > A (p.Val79Glu) of the SERPINA1 gene. Despite this genetic similarity, their clinical courses differed significantly. The patient in case 1 experienced severe illness, while the patient in case 2 presented with milder symptoms.

### 2.1 Case 1

**Fig. 2:**
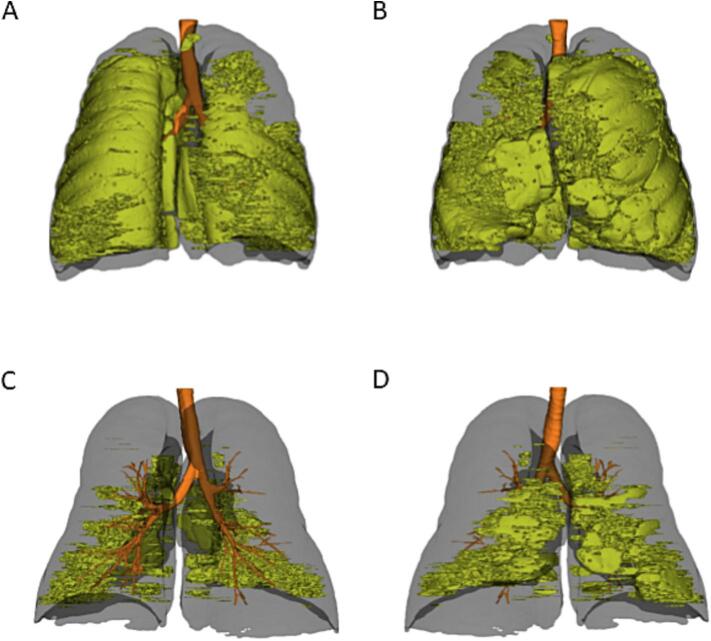
Distinct emphysema presentation in brothers that share a novel identified rare mutation. The results of quantitative analysis of emphysema (YACTA) in eight consecutive thin-slice high-resolution computed tomography (HRCT) datasets with varying emphysema severity and distribution patterns are presented. The emphysema voxel, (−950 HU), is shown in yellow. A and B: Pretransplant HRCT (recorded 01.03.2019) of patient 1 showing severe heterogeneous pulmonary emphysema with an emphasis on the basal lobes. C and D: Chest HRCT showing a basal panlobular emphysema of patient 2 (recorded 27.02.2024). A and C: Coronal frontal view of both patients. B and D: Coronal posterior view.

The older brother, a 66-year-old patient, was diagnosed in 2000 with very severe COPD (GOLD stage IV) and AATD. His comorbidities included arterial hypertension, gastroesophageal reflux, hyperlipidemia, and sigmoid diverticulitis. He has a history of smoking, with a 17-year history, which ended in 2000. He was born full-term, had no history of recurrent lung infections during childhood, and worked as a locksmith throughout his life. He lived in a small town away from busy streets and reported no relevant household smoke exposure. At diagnosis, his AAT serum level was low at 0.32 g/L (≈6.1 μM), while liver enzyme levels were within the normal range (Table 1). Consistently, a series of CT scans showed no pathological changes in the liver, and physical examinations always revealed normal findings, with no evidence of hepatosplenomegaly. The last abdominal sonography in 2023 (without elastography) showed no pathological changes. Until 2015, his treatment regimen included formoterol/beclomethasone, tiotropium bromide, inhalations with 0.9% sodium chloride solution, and weekly Prolastin augmentation therapy. However, by 2015, progressive lung damage was evident, marked by heterogeneous basal emphysema with bullae formation in the middle lobe. In December 2015, due to clinical deterioration, endoscopic lung volume reduction was performed, involving the implantation of five vents in the left lower lobe, which resulted in significant improvement. Unfortunately, in 2019, the vents were removed due to colonization with *Pseudomonas aeruginosa*, despite multiple attempts at eradication with antibiotics. In March 2019, pre-transplant HRCT showed severe heterogeneous pulmonary emphysema with a focus on the basal lobes (Fig. 2A and B). In 2020, a double lung transplant was carried out, leading to improved lung function parameters (Table 2). In 2024, IEF identified one common Z protein and a second protein with similar migration to the M protein. Genomic PCR-based sequencing of the *SERPINA1* gene revealed heterozygosity for the Z allele (c.1096G > A (p.Glu366Lys); rs28929474) and heterozygosity for a novel mutation at exon 2, position c.236 T > A (p.Val79Glu). The sequencing method used did not allow determination of whether both mutations are located on the same allele or on different ones.

**Table 1:** Summary of the two patient's medical history and clinical data at diagnosis of AATD in 2000.

	Case 1	Case 2	Reference values
Sex	Male	Male	_
Age at diagnosis, years	66	58	_
Comorbidities	arterial hypertension, gastroesophageal reflux, hyperlipidemia, and sigmoid diverticulitis	arterial hypertension, hyperlipidemia, hyperuricemia, and obstructive sleep apnea	_
Smoking history	17 pack-years until 2000	10 pack-years until 2000	_
GOLD COPD status	IV	No COPD	_
AAT, g/L (μM)	0.32 (≈6.1)	0.4 (≈7.6)	0.9–2 (≈17–38)
GOT/AST, U/L	21	20	<46
GPT/ALT, U/L	38	42	<50
CRP, mg/L	2.1	2	<5
Hemoglobin, g/dL	14.8	15.5	13–17
Leukocytes, cells/nL	5.02	9.48	4–10
Neutrophils, %	61.2	81.2	50–80

AAT, alpha-1 antitrypsin; AATD, alpha-1 antitrypsin deficiency; ALT, alanine aminotransferase; AST, aspartate aminotransferase; COPD, chronic obstructive pulmonary disease; CRP, C-reactive protein; GOLD, Global Initiative for Chronic Obstructive Lung Disease; GOT, Glutamate-oxaloacetate transaminase; GPT, Glutamate-pyruvate transaminase.

**Table 2:** Lung function values.

Spirometry	Case 1	Case 1	Case 2
	Pre-transplant	Post-transplant	At AATD diagnosis
FVC, L (%)	1.58 (33.23)	4.3 (97)	4.49 (96)
FEV1, L (%)	0.76 (20.9)	3.28 (97)	3.56 (98)
FEV1/FVC, %	48	76	79
RV, L (%)	9.58 (391)	2.51 (99)	2.32 (98)
DLCO, %	29.2	73	75

AATD, alpha-1 antitrypsin deficiency; DLCO, diffusion capacity of carbon monoxide; FEV1, forced expiratory volume in one second; FVC, forced vital capacity; RV, residual volume.

### 2.2 Case 2

The younger brother, a 58-year-old patient, was diagnosed with AATD in 2000 as part of a screening process following the positive genetic test results from his brother. His comorbidities included arterial hypertension, hyperlipidemia, hyperuricemia, and obstructive sleep apnea. He has a history of smoking, with approximately 10 pack-years, and quit smoking in 2000. Additional details revealed that the patient was born at full term not premature, had no history of recurrent childhood infections, and was not exposed to tobacco smoke at home from parents or other relatives. He lived in a small town, far from busy streets, and worked as a locksmith throughout his life. At diagnosis, his AAT serum levels were low (0.4 g/L [≈7.6 μM]), and his lung function values were as follows: forced expiratory volume in one second (FEV1) was 3.56 L (98%), residual volume (RV) was 2.32 L (98%), diffusion capacity of carbon monoxide (DLCO) was 75%, forced vital capacity (FVC) was 4.49 L (96%), and FEV1/FVC was 79% (Table 1, Table 2). Liver enzymes were within the normal range (Table 1). An abdominal ultrasound showed no structural changes to the liver or gallbladder and no evidence of focal tumor lesions. Elastography showed a normal FibroScan value of 6.1 kPa (ref. <7 kPa), but a CAP value of 328 dB/m (ref. <238 dB/m), confirming fatty liver. This condition is currently uder control, and no treatment is indicated. Clinically, the patient reported a worsening of pulmonary symptoms and the presence of recurrent respiratory infections in 2023. In 2024, a HRCT revealed panlobular basal emphysema with bronchopathy and discrete bronchiectasis (Fig. 2C and D). The same year, genomic PCR-based DNA sequencing of the *SERPINA1* gene identified a novel mutation (c.236 T > A (p.Val79Glu)) in heterozygosity and a Z mutation (c.1096G > A (p.Glu366Lys); rs28929474) in heterozygosity. The sequencing method used did not allow determination of whether both mutations are located on the same allele or on different ones. As a result, he started augmentation therapy with prolastin in 2024. His current therapy includes candesartan, bisoprolol, fluticasone/vilanterol, rosuvastatin, allopurinol, inhalations with 0.9% sodium chloride solution.

## 3 Discussion

We report the case of two siblings with the same mutations in the SERPINA1 gene, Pi*Z (c.1096G > A (p.Glu366Lys); rs28929474) and c.236 T > A (p.Val79Glu), causing AATD, but with distinct clinical presentations. Despite sharing the same genotype, only one of the patients experienced severe clinical and pulmonary impairment. Although sequencing covered all exons and adjacent intronic regions, variants in deep intronic or regulatory regions cannot be entirely excluded and may contribute to phenotypic variability between individuals sharing the same coding genotype.

In Case 1, very severe COPD (GOLD IV) was diagnosed at the time of clinical presentation, followed by further deterioration and, ultimately, lung transplantation. Interestingly, when the genetic diagnosis of AATD was made in the second brother, no severe lung disease was evident. Over the course of his clinical evolution, emphysema developed more slowly and was primarily confined to the basal regions of the lungs, with the addition of non-extensive bronchiectasis.

Analysis of serum AAT revealed low circulating protein levels, well below the commonly accepted threshold for severe deficiency (11 μM) in both patients (Case 1: ≈6.1 μM; Case 2: ≈7.6 μM), leading to the genetic analysis of the *SERPINA1* gene. Since the Progenika AAT genotyping kit is limited to the 14 most common allele variants, targeted Next-Generation Sequencing of all SERPINA1 coding exons and exon–intron boundaries was necessary, revealing two AAT variants: the common variant Pi*Z (c.1096G > A (p.Glu366Lys)) and a novel mutation in exon 2 at position c.236 T > A (p.Val79Glu). Interpreting these results can be challenging, because genotyping cannot determine whether both mutations are present on one or two alleles. Although novel long-read nanopore approaches capable of phasing complete SERPINA1 haplotypes have recently been described [21], these remain proof-of-concept methods, are not commercially available, and were not accessible in routine clinical diagnostics at the time these cases were evaluated. However, low AAT serum levels suggest that it is highly likely that both mutations are on separate alleles, resulting in Pi*Z (c.1096G > A (p.Glu366Lys)) and c.236 T > A (p.Val79Glu) genotype. Moreover, IEF identified a common Z protein and another similar to an M protein, but it is unclear if the described mutation causes a ‘pseudo-M mutation’ or mimics an M-protein. As a result, the classical nomenclature for this new variant cannot be applied, which led to the use of the system name c.236 T > A (p.Val79Glu).

Although the IEF analysis showed a second band with migration similar to an M-type isoform, this pattern should not be interpreted as evidence of a functional or normally secreted AAT protein. Several SERPINA1 mutations migrate in the M-range on IEF while still exhibiting marked functional impairment or secretion defects, and M-like variants are known to be difficult to recognize with routine phenotyping unless specifically interrogated by sequencing or refined diagnostic workflows [22,23]. In our cases, serum AAT concentrations (≈6.1 μM and ≈7.6 μM) were within the range typically observed in individuals with Pi*ZZ genotype, suggesting that the p.Val79Glu variant, if in trans with Pi*Z, likely causes a loss-of-function effect comparable in magnitude to *Z*-associated misfolding and endoplasmic reticulum retention [24].

The p.Val79 residue lies within the helix C region of AAT, a structurally sensitive domain involved in proper folding and stability. Substitution of a hydrophobic valine with a charged glutamate may disrupt local packing interactions, promote misfolding, or increase the tendency toward polymerization—mechanisms documented for other pathogenic AAT variants in which residue changes perturb folding pathways and conformational transitions [25,26]. Such variants may retain near-normal electrophoretic mobility yet fail to circulate due to intracellular degradation or polymer accumulation [23]. Therefore, the observed “pseudo-M" migration pattern most likely reflects preserved isoelectric properties rather than preserved biochemical function. This interpretation is supported by the markedly reduced serum AAT levels in both siblings and the severe clinical phenotype in Case 1, and it is consistent with reports of rare SERPINA1 variants that show reduced secretion and intracellular polymerization similar to Z in cellular models [27].

Although both brothers had similarly low circulating AAT levels, lung disease severity in AATD does not always correlate directly with serum concentration, and substantial phenotypic heterogeneity has been reported. Smoking exposure is a major modifier of emphysema risk and can markedly accelerate lung function decline, even at moderate cumulative doses [28,29]. Thus, the older age at diagnosis and longer smoking history in Case 1 likely contributed to his more severe pulmonary impairment despite comparable biochemical deficiency. Additionally, hepatic and pulmonary manifestations arise through distinct mechanisms—protein retention in hepatocytes versus protease–antiprotease imbalance in the lung—so normal liver enzyme values do not exclude advanced lung disease, consistent with findings in AATD cohorts [30].

Although rare genotypes causing severe AATD are more common than expected and often linked to worse clinical features like Pi*ZZ deficiency (c.1096G > A (p.Glu366Lys)), their clinical relevance is often obscured by challenges in routine genotyping, which typically focuses on the most prevalent allelic variants, as well as the complex biochemical properties of AAT [20,31]. Additionally, multiple rare variants have been shown to form intracellular polymers or exhibit secretion defects, reinforcing that severe biochemical phenotypes are not confined to the canonical Z site [27,32].

The limited data on rare *SERPINA1* gene variants makes it difficult to assess whether affected patients are at higher risk of developing AATD-associated diseases. Our case report underscores the challenges of clinically characterizing certain *SERPINA1* variants once identified and raises the question of whether factors beyond genotype contribute to the risk of developing severe AATD. Therefore, future diagnostics should prioritize not only genetic analysis but also the biochemical evaluation of variant proteins, a thorough review of the patient's medical history, and relevant laboratory values. This comprehensive approach could help clarify the underlying etiology and guide treatment. Personalized clinical management, including preventive and therapeutic interventions available for Pi*ZZ patients (c.1096G > A (p.Glu366Lys)) [20,31,33] may then be more effectively tailored to individual cases.

Additionally, our cases highlight the importance of genetic screening for relatives, which can help identify family members with early-stage conditions who may benefit from medical treatment, as well as those without symptoms who could benefit from lifestyle interventions—such as smoking cessation—that are known to modify disease trajectory in AATD [34]. In clinical practice, we recommend evaluating each case individually, considering factors such as genotype, serum AAT levels, clinical symptoms, and (former) smoking habits to assess a patient's risk of developing AATD-related diseases.

## 4 Conclusion

The precise characterization of the clinical profile of rare *SERPINA1* mutations remains challenging due to uncertainties associated with the biochemical and genetic complexities of these variants, as well as limitations in the current diagnostic algorithms. Despite these challenges, the growing body of case reports on rare mutations provides valuable insights into the disease, which could aid in more accurate identification and timely initiation of therapeutic interventions. These case studies highlight the need for a comprehensive approach to diagnosis, including genetic screening, biochemical analysis, and clinical evaluation, to better understand the full spectrum of AATD-related diseases and improve patient outcomes. Early recognition and individualized treatment plans may help mitigate disease progression and enhance quality of life for affected individuals.

## CRediT authorship contribution statement

**Arturo Olivares-Rivera:** Writing – review & editing, Writing – original draft, Visualization, Software, Resources, Project administration, Methodology, Investigation, Funding acquisition, Formal analysis, Data curation, Conceptualization. **Hilal Ersöz:** Writing – review & editing, Visualization, Investigation, Data curation. **Philipp Höger:** Writing – review & editing, Investigation. **Martina Veith:** Writing – review & editing. **Timm Greulich:** Writing – review & editing. **Kai Schlamp:** Writing – review & editing, Resources, Investigation. **Sabina Janciauskiene:** Writing – review & editing, Writing – original draft, Resources, Methodology, Investigation, Formal analysis, Data curation, Conceptualization. **Felix Herth:** Writing – review & editing. **Franziska C. Trudzinski:** Writing – review & editing, Writing – original draft, Visualization, Validation, Supervision, Software, Resources, Project administration, Methodology, Investigation, Funding acquisition, Formal analysis, Data curation, Conceptualization.

## Consent for publication

Written informed consent was obtained from the patients to publish their anonymized information in this article.

## Ethics approval and consent to participate

The patients provided written informed consent for the publication of their anonymized information in this article. Ethical approval was not required, as the University of Heidelberg does not mandate it for individual case reports. This research was conducted in accordance with the principles of the Helsinki Declaration, revised in 2024.

## Funding

The publication of this case was supported by Grifols.

## Declaration of competing interest

AOR has received honoraria for lectures from Grifols and support for attending meetings from CSL Behring.

HE has received support for attending meetings from CSL Behring.

PH has received honoraria for lectures from Grifols and support for attending meetings from CSL Behring.

MV has received financial support for the lab from Grifols.

TG has received grants for the institution from Grifols; consulting fees from AstraZeneca, Berlin-Chemie, Boehringer-Ingelheim, Chiesi, CSL-Behring, Grifols, GSK, Mundipharma, Novartis, Sanofi, and Takeda; honoraria for lectures from AstraZeneca, Berlin-Chemie, Boehringer-Ingelheim, Chiesi, CSL-Behring, Grifols, GSK, Mundipharma, Sanofi, and Takeda; support for attending meetings from AstraZeneca, Berlin-Chemie, Chiesi, CSL-Behring, Grifols, GSK, Novartis, and Sanofi; participation on advisory board from AstraZeneca, Berlin-Chemie, Boehringer-Ingelheim, Chiesi, CSL-Behring, Grifols, GSK, Mundipharma, Novartis, Sanofi, and Takeda.

KS has no conflicts of interest.

SJ has received support for attending meetings from Grifols.

FH has no conflicts of interest.

FCT received payment lectures, or reimbursement of travel expenses from Novartis AG, GlaxoSmithKline, Chiesi, Boehringer Ingelheim GmbH, Grifols and AstraZeneca, CSL Behring, and Grifols Deutschland GmbH.

## Data Availability

The data that support the findings of this study are not openly available due to reasons of confidentiality and are available from the corresponding author upon reasonable request. The complete sequencing of the *SERPINA1* gene is available in the GenBank Nucleotide Repository (accession number: NM_001127701.1). Additionally, the novel variant c.236 T > A (p.Val79Glu) has been deposited in ClinVar under accession number [SCV007334878], to facilitate future diagnosis and research.
